# Paying for Parenthood: Understanding Parental Leave Policies in Infectious Disease Fellowship

**DOI:** 10.1093/ofid/ofad685

**Published:** 2024-01-04

**Authors:** Catherine P Gardiner, Laura Desrochers, Kathleen Finn, Furman McDonald, Michael Melia, Michael Melfe, Wendy Stead

**Affiliations:** Beth Israel Deaconess Medical Center, Boston, Massachusetts, USA; Beth Israel Deaconess Medical Center, Boston, Massachusetts, USA; Tufts Medical Center, Boston, Massachusetts, USA; American Board of Internal Medicine, Philadelphia, Pennsylvania, USA; Johns Hopkins School of Medicine, Baltimore, Maryland, USA; American Board of Internal Medicine, Philadelphia, Pennsylvania, USA; Beth Israel Deaconess Medical Center, Boston, Massachusetts, USA

**Keywords:** ABIM, parental leave, fellowship training, infectious diseases

## Abstract

**Background:**

Many physician trainees plan pregnancy during residency and fellowship. A study of internal medicine program directors (PDs) demonstrated frequent misinterpretation of American Board of Internal Medicine (ABIM) leave policies applied to parental leave. The primary aim was to investigate how infectious disease (ID) PDs interpret current ABIM leave policies.

**Methods:**

We surveyed 155 ID PDs in an online anonymous questionnaire about knowledge of ABIM leave policies and application toward trainee leaves.

**Results:**

Of 155 PDs, 56 (36%) responded to the survey. Nearly 70% incorrectly identified leave limits permitted. A majority mistakenly chose to extend training when a competent fellow was within the allowed duration of leave. PDs reported that the majority of ID trainee maternity/birth parent leaves (60%) were ≤7 weeks and only 7% were ≥12 weeks; 50% of paternity/nonbirth parent leaves were ≤3 weeks.

**Conclusions:**

Surveyed ID fellowship PDs often misinterpret ABIM leave policies and apply policies incorrectly when given sample scenarios..

## Teaser

Misinterpretation and misapplication of American Board of Internal Medicine leave policies have clear implications for trainee family planning. Ambiguity may lead to shortened parental leaves, inappropriate fellowship training extension, and potential lost job and funding opportunities.

## INTRODUCTION

Many physician trainees plan pregnancy during residency and/or fellowship; in one recent survey of trainees at the Mayo Clinic, 40% of respondents reported planning pregnancy during their current residency or fellowship training [1]. Despite the frequency with which physician trainees become parents, graduate medical education programs historically have held inconsistent and ambiguous parental leave policies or have been without policies altogether, lacking firm guidance by their governing bodies until recently [2].

In July 2020, the American Board of Medical Specialties (ABMS) announced that all ABMS member boards with training programs of at least 2 years must allow for a minimum of 6 weeks of time away from training for purposes of parental, caregiver, and medical leave at least once during training, without exhausting all other allowed time away from training and without extending training, effective July 2021 [3]. The Accreditation Council for Graduate Medical Education (ACGME) echoed these recommendations in its institutional requirements, effective July 2022, requiring accredited programs to allow at least 6 weeks of paid parental, caregiver, and medical leave.

Within internal medicine and its subspecialties, program directors (PDs) have 2 official leave policies set forth by the American Board of Internal Medicine (ABIM): the Leave of Absence and Vacation Policy, which previously allowed for 1 month of leave per academic year, and the Deficits in Required Training Time Policy, which PDs and competency committees could apply on behalf of competent trainees with training deficits of 1 month per total training time. In a recent study by Finn et al, internal medicine PDs struggled to correctly apply these leave policies to parental leave [4]. When asked about specific scenarios for structuring leave, 83% incorrectly chose to extend training when residents were within the previously determined leave limits [4]. Because of these findings, in 2020 the ABIM revised its website to clarify the Leave of Absence and Vacation Policy to more clearly define permitted time away from training as “up to 5 weeks (35 days) per academic year,” meaning that a resident in a 3-year internal medicine training program could take leave for up to 105 days without needing to extend training [5]. At the same time, the Deficits in Required Training Time Policy was clarified, outlining an additional 5 weeks (35 days) of leave that could be granted to competent trainees by PDs and competency committees without requiring an extension in training, subject to ABIM review.

**Figure 1.**
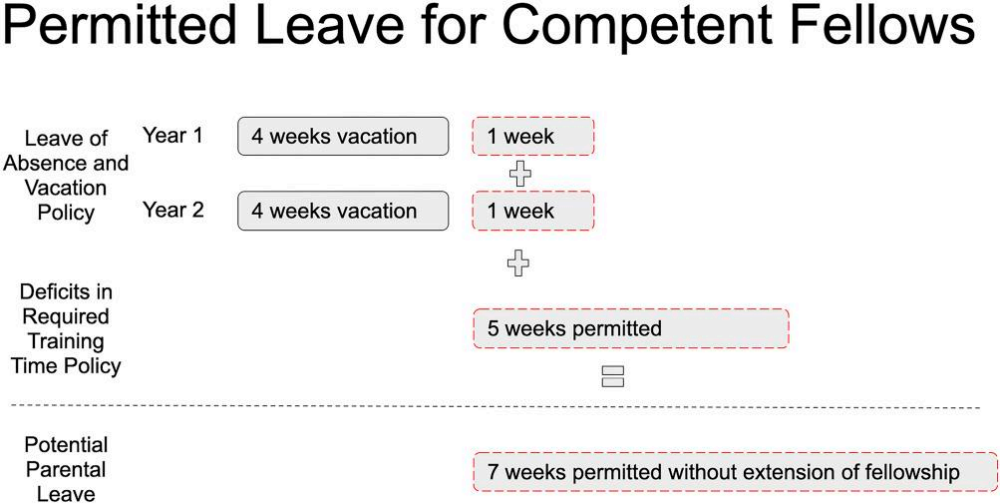
Permitted leave length for clinically competent fellows. Dashed outline indicates additional trainee leave time.

In Figure 1, we summarize the current ABIM leave policies outlining that a clinically competent trainee is allowed 5 weeks (35 days) of vacation or leave annually during training, plus an additional 5 weeks (35 days) under the Deficits in Required Training Time Policy without requiring an extension in training. We illustrate this policy in the example presented here, where in a 2-year infectious disease fellowship training program that grants 4 weeks of vacation annually for its trainees, these policies allow for 7 weeks of additional trainee leave time if a PD invokes the deficits in training policy.

Our study investigated the interpretation and application of the revised ABIM leave policies by infectious disease PDs.

## METHODS

We surveyed 155 infectious disease PDs using an online anonymous questionnaire to characterize their knowledge of ABIM leave policies and application toward trainee leaves of absence. Email addresses were sourced from the Infectious Diseases Society of America’s training PDs Listserv, and the survey was sent 3 times with reminders throughout 2021. The survey tool was adapted, with permission, from a recent study of internal medicine PDs conducted by Finn et al [4]. The survey included questions assessing (1) general PD knowledge of ABIM leave policies and (2) how PDs would apply these policies when provided with case-based examples of fellows requesting various leave lengths, especially with regard to requirements for training extension. Surveys also included program-specific demographic questions about dedicated vacation time, frequency with which fellow trainees utilize parental leave, and average parental leave lengths for birth and nonbirth parents. Respondents were also asked to share the strategies that they utilize to avoid training extensions for clinically competent fellows who may exceed the total leave length allocated by the ABIM.

Data were collected via REDCap, a free and secure web-based application that is HIPAA compliant (Health Insurance Portability and Accountability Act). Approval was obtained from the Beth Israel Deaconess Medical Center’s institutional review board, and the study was deemed to be exempt. Survey data were analyzed by basic descriptive statistics.

## RESULTS

**Figure 2.**
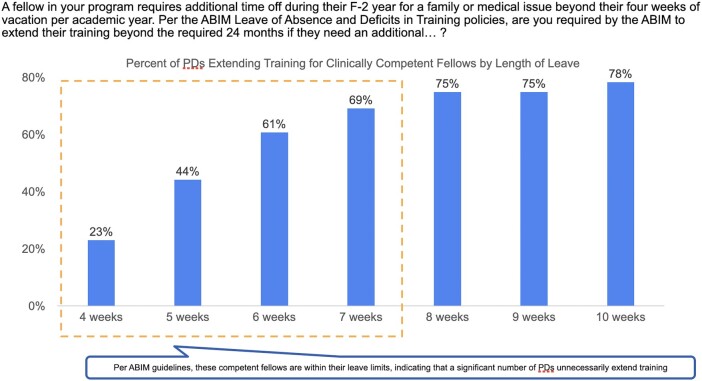
Proportion of program directors extending training by leave duration based on 4 weeks of vacation per year. ABIM, American Board of Internal Medicine; PD, program director.

Of 155 PDs, 56 (36%) responded to the survey. When asked to identify current ABIM leave policies, many respondents (33/56, 59%) did not know or they misidentified the leave limits permitted by the recently revised ABIM policies. When given several case-based examples of clinically competent fellows requesting various leave lengths for family, medical, or parental leave, a substantial majority of PD respondents (36/52, 69%) stated that they would extend fellowship training for a trainee requesting a 7-week leave. Extension of fellowship was suggested by PDs regardless of the reason for the leave request. Even for a leave as short as 4 weeks, nearly one-quarter of PDs (12/52, 23%) stated that they would extend fellowship training. Figure 2 demonstrates the proportion of PDs who would extend leave for a trainee who required varying degrees of additional time off in their F2 year based on a program that offered 4 weeks of vacation per year. As demonstrated by the dashed line, a significant number of PDs would unnecessarily extend a fellow's training even if the fellow leave length is within the 7 weeks permitted by the new ABIM guidelines.

**Figure 3.**
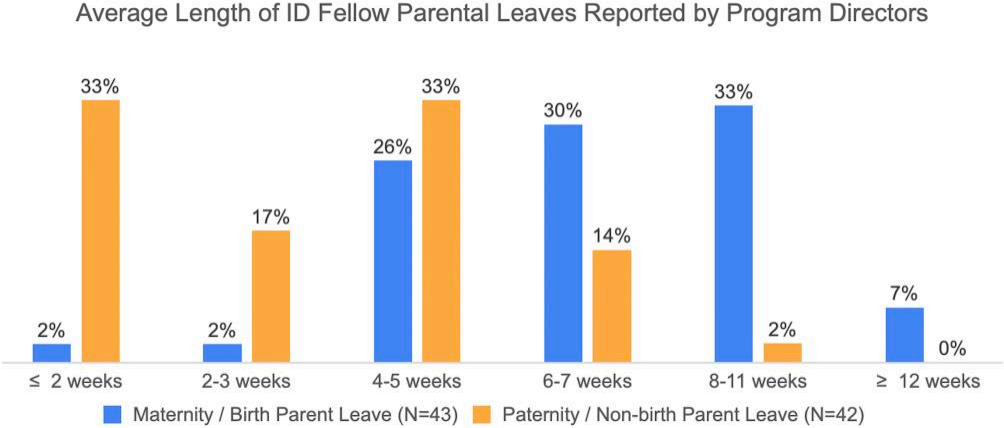
Average length of parental leaves taken in infectious disease fellowship. ID, infectious disease.

Average leave lengths varied widely, with the majority of leaves being ≤7 weeks (60% of birth parent and 98% of nonbirth parent). Most respondents (78%) correctly identified that equal time is permitted for birth and nonbirth parent leave. However, despite this recognition, PDs reported tremendous variation in parental leave lengths between birth and nonbirth parent trainees within their programs. While 70% of birth parent leaves were ≥6 weeks, 83% of nonbirth parent leaves were ≤5 weeks and 50% were ≤3 weeks. Figure 3 demonstrates the average length of parental leaves for birth and nonbirth parents.

PDs reported utilizing various strategies to prevent extending training for fellows taking parental leaves that exceed the limits allowed by ABIM policies, including creation of home electives, having the fellow participate in ambulatory clinics, or rearranging of clinical and research time. Notably 34% (17/50) of respondents reported counseling trainees to take a shorter parental leave and combine it with vacation to minimize disruption to the program and their training.

## DISCUSSION

In July 2020, the ABMS announced that all its member boards with training programs of at least 2 years must allow for a minimum of 6 weeks of time away from training for purposes of parental, caregiver, and medical leave at least once during training, without exhausting all other allowed time away from training and without extending training, effective July 2021 [3]. The ACGME echoed these recommendations in its institutional requirements, effective July 2022, requiring accredited programs to allow a minimum of 6 weeks of paid leave at least once and at any time during an ACGME-accredited program, with at least 1 other week of paid time off reserved for use outside of the first 6 weeks of the first approved medical, parental, or caregiver leave [6]. Note that our survey was administered in spring of 2021, well after the initial ABMS announcement but before the updated ACGME institutional requirements became effective; therefore, PD survey respondents may have been unaware of new ACGME institutional guidance. Of note, these ACGME institutional polices on vacation and leaves of absence are not included in the most recent documents on common [7] or infectious disease–specific [8] program requirements, which PDs often reference for training program guidance; however, with regard to parental, medical, or caregiver leave, all they state is that “each program must allow an appropriate length of absence for residents unable to perform their patient care responsibilities.” Beyond minimum leave length policies, however, infectious disease PDs turn to the ABIM, in its role as the subspecialty certifying board, for the most specific and granular leave of absence guidance.

Despite recent attempts to set minimum trainee leave lengths and ABIM leave policies, we found that a majority of fellowship PDs in infectious disease incorrectly identified ABIM leave length policies and mistakenly chose to recommend extension of fellowship training for competent fellows taking leave. In addition to the impacts of unnecessary training extensions on trainee well-being, there are long-term financial and professional implications of the unnecessary extension of fellowship training programs on fellows requesting leave, including new parents. For example, extended training may force a delayed start date of an attending role, with significant wage losses. Furthermore, if fellowship extension continues beyond the yearly board examination, board eligibility may be delayed, also disrupting career progression and opportunity.

Interestingly, although PDs in our study frequently misinterpreted ABIM leave policies, most correctly identified that equal time is permitted for birth and nonbirth parents. However, in practice, many nonbirth parent leaves were much shorter than birth parent leaves, a finding documented by others and felt to reflect implicit bias minimizing the role of nonbirth parents in early childcare [9, 10]. In addition to excluding nonbirth parents from fully sharing in this crucial time of parent-child bonding, these leave length disparities may establish an early and often perpetuated dynamic by which birth parents (often women) assume the greater burden of childcare from the time of the child's birth, having a potential downstream longer-term impact on their career opportunities as they continue to shoulder a disproportionate amount of dependent care responsibilities. Another possibility could be that some fellows know that they have only 2 years of training and they desire to maximize their clinical time and education.

Although some PDs continue to counsel fellows to take shorter leaves, our study found that many have developed creative strategies for preventing extension of fellowship training for competent trainees who take leaves exceeding ABIM limits. Although some may not recognize this, as medical education leaders of their training programs, PDs have authority to be creative with their program training structure as long as they adhere to ACGME Infectious Diseases Program requirements and ensure that trainees complete the 12 months of clinical training required by the ABIM. A clear and transparent statement from the ABIM would empower PDs to assist trainees in planning parental leaves—one stating that, as long as program competency committees deem trainees competent, PDs can creatively craft effective learning experiences that permit adequate leaves and still allow trainees to graduate on time. It is likely that PDs who do not extend training (up to 25% in our study) are already using creative strategies such as flexible elective time to meet clinical and competency requirements. An online leave calculator whereby PDs could enter program vacation time, clinical time, and leave length requests to determine whether they are exceeding ABIM limits might also be helpful. A calculator might provide additional transparency for trainees to allow for planning and advocacy to their programs if the policy is not fairly applied. Moreover, PD-specific education at conferences or meetings may help raise awareness of current leave policies and empower PDs to adopt strategies to avoid extension of fellowship and to advocate for greater equity in leave lengths for birth and nonbirth parents. Finally, PD-specific education at conferences or meetings may help raise awareness of current leave policies as well as wider adoption of strategies to avoid extension of fellowship.

Our study has several limitations. Our response rate was quite low (36%). Only PDs from infectious disease fellowship were surveyed. We did not survey other internal medicine subspecialty program leaders. Yet, it is likely that these issues arise in other medical subspecialty training programs as well, given that the same ABIM leave policies apply. Furthermore, our survey was administered at a time of policy evolution regarding new parental leave minimums put forth by the ABMS and ACGME and may reflect policies that PDs would otherwise change in the future. However, because PDs look to the ABIM, in its ultimate certifying role, for the most specific and relevant leave length policies, these changes did not likely affect survey results.

## CONCLUSIONS

The recent ABIM revisions to clarify trainee leave policies continue to be misinterpreted by infectious disease PDs, potentially resulting in shortened parental leaves and unnecessary training extensions. While the ACGME and various specialty licensing boards continue to promote standards for minimum trainee parental leave lengths for US physician training programs, at the institutional level, PDs must ensure that they are correctly and effectively applying current ABIM policies and executing the power that they have as program education leaders to help structure leaves that best meet the needs of their trainee parents. Future work to advocate for trainee parents should extend beyond PD knowledge but also create transparency for trainees to make informed decisions regarding family planning.

## Notes

***Patient consent statement.*** Our study did not include factors necessitating patient consent. The design of the work was reviewed by our institutional review board and deemed exempt. This study was unfunded.
